# Restoration and repair of Earth's damaged ecosystems

**DOI:** 10.1098/rspb.2017.2577

**Published:** 2018-02-28

**Authors:** Holly P. Jones, Peter C. Jones, Edward B. Barbier, Ryan C. Blackburn, Jose M. Rey Benayas, Karen D. Holl, Michelle McCrackin, Paula Meli, Daniel Montoya, David Moreno Mateos

**Affiliations:** 1 Department of Biological Sciences and Institute for the Study of the Environment, Sustainability, and Energy, Northern Illinois University, DeKalb, IL, USA; 2 Department of Biological Sciences, Northern Illinois University, DeKalb, IL, USA; 3 Department of Economics and Finance, University of Wyoming, 1000 E University Ave, Laramie, WY, USA; 4 Fundación Internacional para la Restauración de Ecosistemas, Madrid, Spain; 5 Departamento de Ciencias de la Vida, Universidad de Alcalá, Alcalá de Henares, Spain; 6 Environmental Studies Department, University of California, Santa Cruz, CA, USA; 7 Baltic Sea Centre, Stockholm University, Stockholm, Sweden; 8 Natura y Ecosistemas Mexicanos AC, Mexico DF, Mexico; 9 Centre for Biodiversity Theory and Modeling, Station D'Ecologie Experimentale du CNRS, Moulis, France; 10 Centre INRA de Dijon, Dijon Cedex, France; 11 Basque Center for Climate Change – BC3, Bilbao, Spain; 12 IKERBASQUE, Basque Foundation for Science, Bilbao, Spain

**Keywords:** restoration, recovery, disturbance, resilience

## Abstract

Given that few ecosystems on the Earth have been unaffected by humans, restoring them holds great promise for stemming the biodiversity crisis and ensuring ecosystem services are provided to humanity. Nonetheless, few studies have documented the recovery of ecosystems globally or the rates at which ecosystems recover. Even fewer have addressed the added benefit of actively restoring ecosystems versus allowing them to recover without human intervention following the cessation of a disturbance. Our meta-analysis of 400 studies worldwide that document recovery from large-scale disturbances, such as oil spills, agriculture and logging, suggests that though ecosystems are progressing towards recovery following disturbances, they rarely recover completely. This result reinforces conservation of intact ecosystems as a key strategy for protecting biodiversity. Recovery rates slowed down with time since the disturbance ended, suggesting that the final stages of recovery are the most challenging to achieve. Active restoration did not result in faster or more complete recovery than simply ending the disturbances ecosystems face. Our results on the added benefit of restoration must be interpreted cautiously, because few studies directly compared different restoration actions in the same location after the same disturbance. The lack of consistent value added of active restoration following disturbance suggests that passive recovery should be considered as a first option; if recovery is slow, then active restoration actions should be better tailored to overcome specific obstacles to recovery and achieve restoration goals. We call for a more strategic investment of limited restoration resources into innovative collaborative efforts between scientists, local communities and practitioners to develop restoration techniques that are ecologically, economically and socially viable.

## 1. Introduction

The pace of ecosystem destruction from anthropogenic and natural impacts is rapid, with billions of US dollars spent annually to restore damaged ecosystems [1,2]. As most of the Earth is impacted either directly or indirectly by people [3], restoration has emerged as one of the most important tools to stem the biodiversity crisis and repair damaged ecosystems [4]. Ecological restoration projects have been carried out for decades using a range of strategies and meeting with a wide range of successes and failures. Whereas the goals of restoration vary and are highly debated [5,6], most restoration projects aim to assist the recovery of key ecosystem attributes towards a reference model [7,8].

The science of ecological restoration, however, is relatively young and has yet to fully take advantage of the potential to look for general patterns across multiple restoration projects to inform our understanding of ecosystem resilience, recovery and functioning. Studies of the influence of restoration efforts on ecosystem recovery and rates are dominated by projects that monitor single sites and are carried out over short periods of time [9]. There has been work to understand how completely and how fast specific ecosystems recover after specific disturbances [10–16]. Yet the lack of research on general recovery patterns across ecosystems makes rigorous tests of theory about restoration trajectories and evaluation of strategies to maximize restoration outcomes difficult.

**Figure 1.**
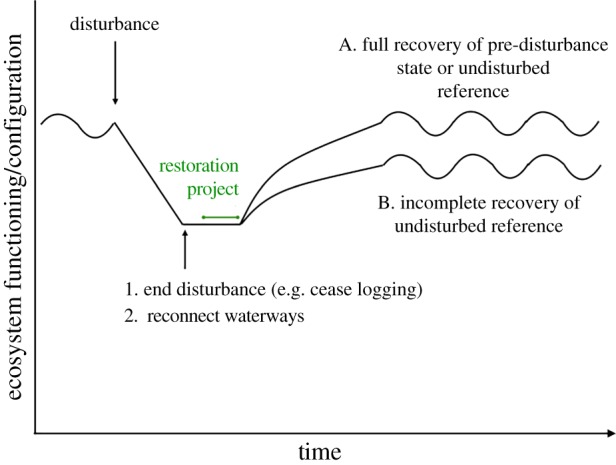
Conceptual figure of two different ecosystem recovery trajectories following the end of a disturbance, according to ecological restoration theory. In trajectory A, the ecosystem fully recovers to the pre-disturbance or undisturbed reference state. Trajectory B would exhibit a lower recovery rate, as is evidenced by its shallower slope following restoration, and would exhibit a negative response ratio (recovery completeness) as it did not reach the pre-disturbance or undisturbed control level. There were two ways to end the disturbance and initiate the recovery process in our analysis: either end the disturbance or restore hydrology. Examples of active restoration are detailed in the electronic supplementary material, table S1. We emphasize that this figure conveys conceptual information and that actual recovery trajectories may take on nonlinearities not depicted here. (Online version in colour.)

Here we present a meta-analysis of 400 studies and 5142 response variables—the variables researchers measured—to document ecosystem recovery from large-scale anthropogenic disturbances (agriculture, eutrophication, hydrologic disruption, logging, mining and oil spills). The resulting studies catalogued recovery after disturbances globally (electronic supplementary material, figure S1) with a combination of actions to end the disturbance, which we define as passive recovery, and to increase the rate and extent of recovery of damaged ecosystems after the disturbance ceased, which we term active restoration (figure 1; electronic supplementary material, table S1), consistent with terms prevalent in the restoration literature [8,17,18]. Our main objectives were to (i) calculate the extent (completeness) and rate of recovery in damaged ecosystems globally and compare these across ecosystems, disturbances, metrics and organism types, and (ii) compare recovery completeness and rates in actively restored versus passively recovering ecosystems.

## 2. Material and methods

We collected data with a standardized search of two literature databases (Web of Science and the first 10 pages or 50 accounts on Google Scholar, whichever came first). We used the search string ‘disturbance type’ (listed in the previous paragraph) AND ‘recov*’ OR ‘restor*’ OR ‘resilience’, and searched abstracts for relevance, finding 972 manuscripts from 1900 to May 2013. The disturbance is defined as an event with the potential to impact ecosystems to which researchers measured a response. We estimated how close response variables were to either a pre-disturbance (*n* = 1092 variables) or nearby undisturbed reference system (*n* = 4001 variables), through measuring response ratios (to indicate recovery completeness; hereafter termed recovery completeness) and the rate of recovery (percentage improvement per year; figure 1). For the 49 variables that had both types of references (i.e. those studies that used before-after-control-impact designs), we used the pre-disturbance reference to calculate recovery completeness. This yielded 400 studies that were identified by the lead author (electronic supplementary material, figure S11).

We considered two different variables to measure restoration success: recovery completeness and recovery rate. Recovery completeness is measured as the response ratio, a commonly used metric in meta-analysis [19–21], and we use the term recovery completeness to refer to our response ratio calculations throughout the main text, while using response ratio when referring to this metric in reference to meta-analysis generally. While other metrics are also commonly used in meta-analysis, those metrics require variance or sample size data, which was not commonly reported in our dataset (electronic supplementary material).

We used the following formula to calculate recovery completeness:
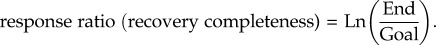
With End representing the response variable's value at the end of the study and Goal representing the undisturbed reference value.

We calculated recovery rates with the following formula:
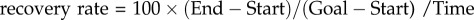
With Start representing the response variable's value at the start of a given study, End representing the response variable's value at the end of the study, Goal representing the undisturbed reference value, and Time representing the number of years since the disturbance ended. We calculated recovery rates rather than static recovery times because recovery times do not factor in differences in disturbance magnitude and thus cannot be accurately compared.

For the meta-analysis, we used general linear mixed models to test the effects of passive recovery versus active restoration, as well as the relative impacts of different types disturbances, ecosystems, response metrics, trophic levels and organism types on recovery completeness and recovery rate. We found ecosystems had more complete and faster recovery with decreasing latitudes (i.e. near the tropics), so we included latitude as a random factor in our analyses (electronic supplementary material).

### (a) Datasets

Our data analysis involved two datasets. The first dataset contains data only from studies in which analyses of both an actively restored site(s) and passively recovered site(s) in a single study were performed, hereafter referred to as the passive–active dataset. Because this first dataset represented a very small portion of the total number of studies (8.5%, 436 out of 5142 response variables, 16 studies) from our literature search, we also used a second dataset containing data from all studies in the literature search, hereafter referred to as the all-studies dataset. We removed category levels with fewer than six studies in our datasets on a per model basis to keep a minimum sample size.

### (b) Models

We used the multivariate model function *rma.mv* from the *metafor* package to construct general linear mixed models [22] in R v. 3.0.1 [23]. Recovery rate values were inverse hyperbolic sine transformed prior to analysis to improve normality and homoscedasticity. Outliers remained after the transformation, and we identified and removed these outliers via the interquartile range method [24]. The study was included as a random factor to account for non-independence of multiple response variables in a single study. The absolute value of latitude also was included as a random factor to account for climatic effects dependent on geographical location. Because our models were random-effects models, tests for publication bias via funnel plots or other means would not be instructive because they assume a fixed-effect model structure [25].

**Figure 2.**
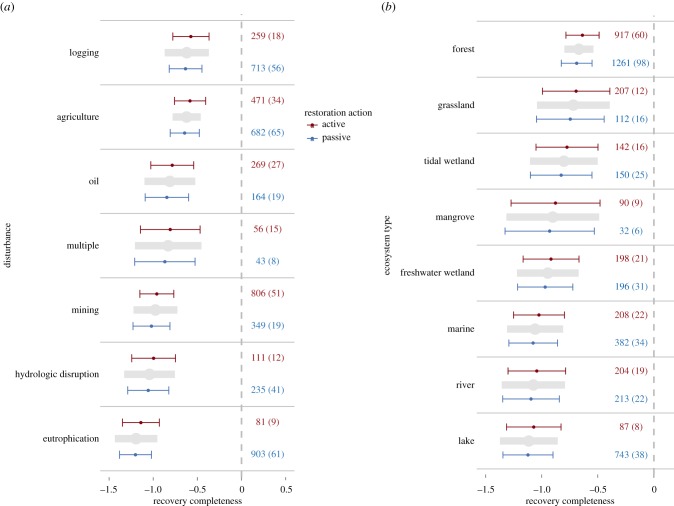
Recovery completeness ± 95% confidence intervals of variables categorized by (*a*) disturbance and (*b*) ecosystem type. Blue lines indicate response variables undergoing passive recovery, whereas red lines depict actively restored variables. Complete recovery is achieved when error bars overlap zero, which is represented by the dashed lines in the panels. Grey bars in the middle of each category are predicted values ± 95% confidence intervals for each independent variable without including recovery type in the models. Data are ordered by the grey bars closest to complete recovery (top) down to the furthest from recovery (bottom). Numbers next to each line are the number of response variables used to model that category and the number of studies from which those response variables were calculated in parentheses. Note that in some cases the same citation could fall in two different categories.

**Figure 3.**
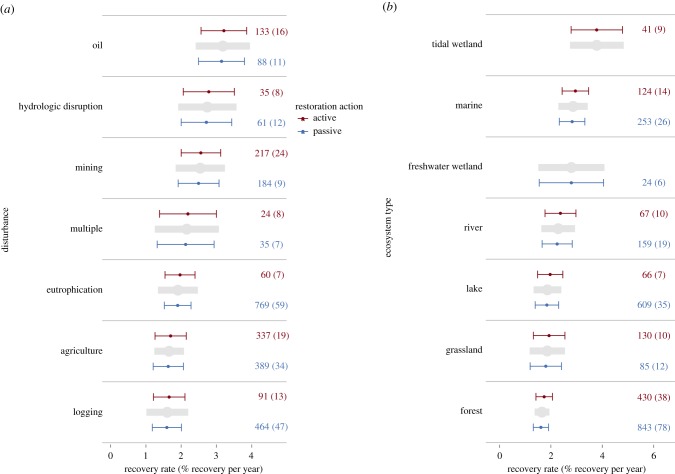
Recovery rate (percentage improvement per year) ± 95% confidence intervals of variables categorized by (*a*) disturbance and (*b*) ecosystem type. Data are ordered by the grey bars with the fastest recovery rate (top) down to slowest recovery rate (bottom). See legend of figure 2 for interpretation of numbers next to lines.

We created three sets of model structures to address our questions about the effects of restoration (electronic supplementary material, table S4). The first model structure included one of the five categorical variables (type of disturbance, ecosystem, metric, trophic level and organism; see electronic supplementary material, table S3) and the additive effect of recovery type as moderator variables. The results of these models are shown in figures 2 and 3, and electronic supplementary material, figures S2, S3 and S6. The second set of models included only one of the five categorical variables listed above as a main effect. The third model structure was our most inclusive and included only recovery type as the main effect (electronic supplementary material, figure S10).

We used the first model structure with both the all-studies and passive–active datasets to build models with recovery completeness and recovery rate as dependent variables. We also used the first model structure with the all-studies dataset to build models with disturbance magnitude as the dependent variable. Because the data requirements were relatively high for the finer scale analysis, we only used the all-studies dataset to build models with recovery completeness and recovery rate as dependent variables for those model structures. We used the second model structure to build model sets for both the all-studies and passive–active datasets for the purpose of determining the ‘average’ effects of the five categorical variables listed above for recovery completeness, recovery rate or disturbance magnitude as dependent variables (grey bars in figures 2 and 3; electronic supplementary material, figures S2, S3, S6 and S7). We used the third model structure to build model sets for the all-studies to determine the relationship between active and passive recovery with no other moderating variables and recovery completeness and recovery type as dependent variables. To determine whether moderators such as recovery type had a significant effect on the dependent variable, we ran likelihood ratio tests between nested models.

Because each model was structured differently and required a minimum number of studies per category, we ran each model with a different set of response variables (electronic supplementary material, table S4), precluding the use of model averaging. Moreover, as is common in ecological meta-analyses [26], the data gleaned for our study were highly variable even among similar categorical variables. Therefore, we present the modelled recovery completeness and recovery rates and their confidence intervals as broad patterns, rather than testing for which categorical variables were most important in influencing recovery completeness and recovery rate, which was not possible with our dataset.

## 3. Results and discussion

Overall, we found that ecosystems are not fully recovering from large-scale disturbances; that recovery rates varied among ecosystems, disturbances, recovery metrics and organisms; and that active restoration did not consistently speed or achieve more complete recovery than letting ecosystems recover without additional assistance. We did find positive recovery rates in all cases, which means that although systems are not recovering completely, they are regaining some of their biodiversity and ecosystem functioning following disturbances.

### (a) Extent of recovery

At the start of recovery, ecosystem response variables in our analysis had median values that were 10% of their reference conditions and ecosystems rarely recovered to reference conditions across ecosystem or disturbance types (figure 2; electronic supplementary material, figure S2), though forests and grasslands were the closest, and lakes and rivers were the furthest, from complete recovery (grey bars in figure 2*a*). Ecosystems were furthest from complete recovery following eutrophication and closest to recovery following logging (grey bars in figure 2*a*), a counterintuitive result considering the turnover time for the dominant organisms in aquatic systems are much shorter than those in forests [27], which should theoretically allow aquatic systems to respond more quickly to improved conditions [28]. One potential explanation is that aquatic systems undergoing eutrophication often experience many other concurrent pressures and non-point sources of pollution that may prevent full recovery or even a complete end to disturbance in those systems [15].

It could be that the lack of complete recovery is due to an insufficient amount of time to detect ecosystem recovery. However, we found recovery completeness did not vary with time since restoration (*p* = 0.73), which was surprising. A previous study found a similar near-universal lack of complete recovery using a subset of the current dataset (*n* = 89 studies versus the 400 in the current study) and when viewing recovery through an ecosystem services lens [21]. Our results expand those findings to a broader range of ecosystems and geographies, and, together with previous work [29–32], suggest the majority of ecosystems have not yet recovered fully following disturbance and may not in the future. Thus, restoration should not be considered a substitute for conservation, which is a key strategy to ensure sustained support of biodiversity and delivery of ecosystem services in the future.

Restoration ecology is a nascent science; the lack of consistent complete recovery in our study suggests developing the tools to have a high success rate in every ecosystem remains an important priority. Even those methods known to work in particular ecosystems have the potential to be refined and tailored to local site conditions. Hence, we encourage practitioners, scientists and funders to use the growing knowledge of ecological interactions and processes, as well as restoration studies elsewhere, to collaboratively develop and test innovative restoration approaches that are appropriate for local ecological, social and economic conditions.

### (b) Recovery rates

Consistent with previous findings [21], recovery rate was estimated to be positive in all cases and categories, indicating that even though complete recovery is rare, ecosystems show improved biodiversity and ecosystem functioning compared with degraded sites, and are progressing towards reference conditions with time (figure 3; electronic supplementary material, figure S3). Most recovery rates were between 1% and 10% recovery per year (median = 2.9%), while some categories showed even faster recovery. Such rates could lead to full recovery in a human lifetime in the case of ecosystems only pushed slightly away from reference conditions, or it could take millennia for full recovery of ecosystems pushed well away from their reference conditions. The median value of ecosystem attributes at the outset of recovery was 10% of that of the reference values, indicating recovery should be achievable for most damaged ecosystems within decades, assuming a constant recovery rate through time. However, we found faster recovery for variables with shorter times since recovery began (electronic supplementary material, figure S4), with recovery rate decreasing by 0.026% per year since recovery began. Given that half our dataset had no more than 10 years to recover, the recovery rates we estimate may be optimistic, especially for systems in the later stages of recovery. Recovery trajectories may slow in later stages of restoration, while the species, functions and interactions that are most difficult to restore remain elusive. Nonlinear recovery trajectories could explain why we find potential for complete recovery, but our results rarely show it.

Indeed, recovery trajectories can be nonlinear for ecosystems recovering from major disturbances [33]. For example, before restoration is undertaken, ecosystems may be stuck in an alternate stable state, with hysteresis preventing linear recovery [33], and in systems with episodic recruitment, recovery will necessarily be punctuated [34]. Restoration may change such nonlinear trajectories at multiple points to influence progress towards reference conditions, but our data cannot examine these trends as we only have two points in time during recovery. More research on the role of restoration in influencing the shape of recovery trajectories could help address this.

Wetland and marine systems showed the fastest recovery rates (grey bars in figure 3*b*), mainly following oil spills and hydrological disruptions (grey bars in figure 3*a*) and lakes and forests had the slowest recovery rates (grey bars in figure 3*b*). Logging and agricultural disturbances produced the slowest recovery rates, potentially because of the time it takes to rebuild ecosystems that are completely cut down and/or replaced by farmland in comparison with disturbances that leave systems altered but relatively intact.

Among primary producers, algae showed the fastest recovery rates, and submerged aquatic vegetation showed the slowest rates, probably a function of many studies measuring aquatic vegetation tracking responses of slowly recovering lakes. Grasses and herbs tended towards faster recovery than trees/shrubs, which could be due to their faster growth rates, lifespans and life cycles. Invertebrates were associated with slower recovery rates than birds and fish, though the difference was rather minuscule (electronic supplementary material, figure S3). Recent modelling suggests that ecosystem recovery is best achieved when predators and prey are restored together [35], but our results show there may be differences in their recovery rates because higher trophic levels were associated with faster recovery than lower ones (electronic supplementary material, figure S3).

### (c) Recovery progress and restoration goals

Restoration efforts often aim to recover the biodiversity and ecosystem functions that were lost with disturbance [8]. The fact that our study often finds a lack of complete recovery but progress towards a reference, suggests that restoration goals may need to be modified so they are more realistic to actual ecosystems' ability to withstand/recover from damage. Many in the field of restoration ecology have called for a move away from historical reference goals to consider other endpoints that might be more attainable, such as contemporary reference sites that exist today but have not experienced the focal disturbance [36–39]. Our dataset reflects that shift, with 79% of variables compared against contemporaneous rather than historical reference values. Nonetheless, even those that used existing reference sites rarely fully recovered, so a further shift in the expectations of restoration outcomes may be necessary. For example, targets for the minimum amount of biodiversity and ecosystem functions that will meet the needs of the species and people at a given site may be more realistic [6].

The target of restoration is highly debated, and changes in both the conceptual literature and in practice. Many contemporary ecosystem recovery goals go beyond the two we investigate here, speeding recovery or achieving more complete recovery than passively recovering systems (figure 1), and may instead seek to recover specific species or ecosystem functions, enhance human livelihoods or achieve other societal goals [5,40]. Some have also argued that goals reflecting potential future ecosystem conditions might be more realistic given the realities of the extent of human land transformation and climate change [6,40], though this proposal has been contentious [41]. Still, most restoration projects aim towards a historical or contemporary reference model [8], which is the most tractable approach from a regulatory perspective. We concur with Higgs *et al.* [5] that historical or reference systems should serve as a guide, but that, globally, a variety of types of restoration goals (contemporaneous reference sites, a target level of functioning/biodiversity and forward-looking) are necessary to ensure they are attainable and that potential biodiversity and ecosystem service gains are maximized.

### (d) Active restoration versus passive recovery

Active restoration (red lines in figures 2 and 3; electronic supplementary material, figures S2, S3, S6 and S10) was not associated with more complete recovery or faster recovery than passively recovering systems (blue lines in figures 2 and 3; electronic supplementary material, figures S2, S3, S6 and S10). We attempted to reduce the variation between studies by using various categories (ecosystem, disturbance, metric type, etc.) and comparing active restoration versus passive recovery in only those categories and still rarely found benefits of restoration. Moreover, the results from our analysis on those studies that did direct comparisons showed similar patterns (electronic supplementary material, figure S6). These results underscore prior research, which has reported similar results in single ecosystems [12,13,15,16], and extend them to ecosystems globally.

One potential explanation for why we did not find consistently faster or more complete recovery in actively restored ecosystems is that managers may correctly choose which ecosystems are not recovering on their own and require active restoration to improve recovery outcomes relative to passively recovering systems, resulting in the similar rates and levels of recovery found here. Such choices would mean that actively restored sites begin with slower rates/levels of recovery than those that are passively recovering. Then, as restoration proceeds, recovery rates and levels speed up to approximate, but not exceed, recovery rates and levels in passively recovering systems. We cannot test this with our dataset given how few studies (*n* = 16 studies and 436 response variables from the passive–active dataset) compared both passive and active restoration strategies in the same system, which highlights the need for future studies to do so.

We explored three other explanations for why we rarely found overall value added of active restoration across multiple ecosystems. First, though meta-analysis is the best tool to look for general patterns across multiple studies [26,42], the studies included in our analysis may be too disparate for comparison. For example, passive recovery in a grassland after agriculture might not be comparable to active restoration in a forest after agriculture. We did not find, however, that active restoration accelerates or achieves more complete recovery even when including the few studies that compared restoration to passive recovery in the same ecosystem after the same disturbance in a single site (*p* > 0.30 for all comparisons; electronic supplementary material, figure S6). Second, we explored the possibility that sites selected for active restoration might have different initial conditions or ‘disturbance magnitudes’ (i.e. how far ecosystems were pushed from reference conditions when disturbed) than passively recovering ecosystems. Instead, our data show that ecosystems that are actively restored were less disturbed than passively recovering systems (*p* < 0.001; electronic supplementary material, figure S7). Furthermore, we looked to see if variables that had recovered, which we defined as those having a response ratio within 0.05 of completely recovered (ratio = 0) or higher, had different disturbance magnitudes than those variables not recovered, but found no differences (*p* = 0.46; electronic supplementary material, figure S8). Third, we investigated whether actively restored sites had less time to recover than passively recovering sites and found actively restored sites to have three or fewer years to recover on average (*p* < 0.001; electronic supplementary material, figure S9). Given that around half of the variables had 10 or years less to recover, this difference in recovery time could be driving the lack of difference between actively restored and passively recovering sites. However, it is unlikely this is the only explanation for similar rates and levels of recovery and over 2500 response variables in our study had longer to recover.

Although active restoration may be necessary to reverse ecosystem degradation, our results imply that it could be applied more effectively, especially given that restoration can sometimes be ineffective or even hinder recovery [11,43–45]. Letting ecosystems repair themselves in many cases may be the most effective restoration strategy—a counterintuitive yet critical finding that could help society allocate restoration funds more efficiently in the future. To identify where and when active restoration will be most effective, restoration practitioners should, where possible, build formal or informal experimentation into their restoration design [46,47], with one treatment being passive recovery. Another potential option is to give ecosystems some time to recover passively before undertaking restoration to gauge ecosystems' natural ability to recover, an approach that has been adopted in the Forest Code in Brazil [48]. These suggestions may be challenging to implement, given that many land managers have specific mandates, limited budgets, short time frames and/or strong inclinations to actively restore. We do not think active restoration should be avoided based on our findings, but rather recommend that restoration strategies be tailored more closely to overcome the specific barriers to recovery in individual sites.

Although many studies separate active restoration from passive recovery, the distinction between them can be blurry, particularly when ending the disturbance takes significant human input, such as the removal of dams. Accordingly, it is best to view both removing the disturbance and subsequent restoration along a continuum of restoration approaches that distinguishes the effort and resources needed to achieve various restoration outcomes [8]. We were unable to construct and analyse a continuum of restoration effort needed for the various interventions because data on person-hours, cost and resources for each project were unavailable. Future studies that quantify the relative biodiversity and ecosystem services gained per resources spent (e.g. [49]) will be critical to ensuring restoration resources are allocated effectively.

## 4. Conclusion

Restoration ecology is a rapidly developing science, especially as the Earth has undergone dramatic changes that have brought an even greater need to restore damaged ecosystems. With this need have come international and national pledges to restore ecosystems, such as Aichi Target 15 to restore at least 15% of damaged ecosystems by 2020. Based on our results, we recommend the following steps to achieve these targets. First, the goals of specific restoration projects must be clearly articulated so appropriate methods can be selected and their efficacy in achieving desired outcomes evaluated. Second, passive recovery should be considered as a potentially cost-effective option for ecosystem recovery. Third, if rates of passive recovery are insufficient to achieve project goals, then active restoration strategies should be tailored to the local ecological and socioeconomic conditions; these strategies should ideally be compared to a passive restoration approach to help inform future efforts. This multi-step approach will require additional and more strategic investment in restoration to provide the innovative developments needed to meet the ambitious goals being set out by international, national and local communities. Large government and industry partnerships with scientists, local communities and stakeholders (such as those that occurred to send astronauts to the moon and those currently proceeding for cancer research) will be critical to achieving these goals.

## Supplementary Material

Electronic Supplementary Material

## Data accessibility

Data for this research is available from the Dryad Digital Repository (doi:10.5061/dryad.rj849k6). Code for this research is available on the corresponding author's GitHub website (https://github.com/DocHPJones/Restoration_Database).
