# Supplementary material for: Restoration and repair of Earth's damaged ecosystems
Source: Proc Biol Sci. 2018 Feb 28;285(1873):20172577. doi: 10.1098/rspb.2017.2577 (PMC5832705; doi:10.1098/rspb.2017.2577)
Supplement: Electronic Supplementary Material [file rspb20172577supp1.pdf]

Supplementary methods

We extracted graphical data with the freely available program Data Thief (http://datathief.org/) and from tabular data from manuscripts. If authors separated out the reporting of similar response variables (e.g. species richness of the same taxa but in different locations), we maintained separate reporting of those variables with the assumption that authors had valid reasons for reporting multiple variables except where author reasons for separating similar response variables was unwarranted for our level of analysis.

Data quality was ensured in multiple ways. Each person who entered data was trained by the lead author with three manuscripts to ensure accurate numbers were entered and accurate categorizations were made. Data sheets were restricted so that only valid categories would be entered. The lead author met with data enterers weekly to bi-weekly throughout the data collection process to answer questions about data entry and spot-checked data entered throughout the process to ensure accuracy. Lastly, the lead and second authors checked each category assigned per study for accuracy before the data were analyzed. We did not perform an inter-rater agreement statistical test because those are for subjective data sets and ours was objective (reporting numbers from studies).

We combined the data found in the literature search with databases from Moreno Mateos et al. [1], Rey Benayas et al. [2], and Meli et al. [3], resulting ultimately in 400 studies included in our analyses. Studies used field-based measurements to assess ecosystem recovery of various response variables after disturbances. Response variables used to assess recovery were those study authors measured (see Table S2 for examples).

Response ratio calculations are undefined for variables that have end or goal value of zero, despite such response variables being valid data points. A common way to include such data in meta analysis is to add a minimal value to the End and Goal values for the response ratio [1, 2, 4]. We added 0.05 to the End and Goal values of response variables that had a zero End or Goal value and compared those model results to model results excluding response variables with zero values completely. Finding no qualitative difference between findings other than increased variance when including response variables with zero values, we report results including those variables with zero values. Disturbances had contrasting effects on response variables; for example, agriculture could increase the value of invasive species richness/abundance compared to reference or pre-disturbance conditions but decrease the value of native species richness. For consistency in interpreting disturbance magnitude and recovery completeness, as is common in other restoration meta analyses [1, 2], we reversed the sign of recovery completeness for variables that increased under disturbance. In reporting recovery completeness, categories are considered completely recovered if their error bars overlap 0, below complete recovery if they are less than 0, and higher than the reference ecosystem if they are above 0 (more than fully recovered).

Additional model analyses. We investigated the effect of the number of years since recovery began on recovery rate (preliminary analysis indicated the time since restoration started variable did not have an effect on recovery completeness) with a model structure including the number of years since restoration started and recovery type as both additive

effects and as an interactive effect (Fig. S9). We also investigated what effect the year in which a disturbance ended had on recovery. The model structure for this analysis included whether the disturbance ended before 1970 or after as a moderator variable with recovery completeness and recovery rate as dependent variables (Fig. S5).

To investigate potential differences in disturbance magnitude (calculated as [Goal-Start]*100) for actively restored and passively recovering variables we created models where ecosystem and disturbance type were moderators and disturbance magnitude was the dependent variable (Fig. S7). We did a similar model to look at disturbance magnitude for response variables that completely recovered (defined as recovery completeness $\geq$ 0.95) and those not recovered, by modeling recovery state (recovered or not) as a moderator and disturbance magnitude as the dependent variable (Fig. S8). To investigate potential differences in the time since restoration started between sites where active or passive recovery occurred, we created a model with recovery type as the moderator and time since restoration started as the dependent variable (Fig. S9). The random effects structure for all of the additional models included the absolute value of latitude and the study, which are the same random effects included in our main models described above.

We created all figures with the *ggplot2* package [10] in R 3.0.1 [6]. Code for models and figures will be available on the lead author's Github website.

Model weighting. The data necessary to determine variance were not available for the majority of our response variables (24.4%), which is common in ecological meta analyses [1, 2, 11]. When the ratio of among-study variation to within-study variation is very high, meta analysis theory suggests that the parameter estimates of a weighted random-effects model is nearly the same as those from an unweighted model [9, 11, 12]. However, we do want to account for the among- and within-study variance components in our estimates of confidence intervals. We used the subset of studies that reported variances or the information to calculate variances and computed the I^2 index for each response variable. The values of the I^2 index were over 95%, which suggests that among-study variation accounted for the vast majority of the total variance and that the weights across effect sizes would be very similar. To account for this variation and get accurate standard error estimates from our models, we set variance ("vi" in the *rma.mv*() call) to 1 for all data. We then used this variance to weight the mixed meta analytic models.

Categorical variables. See Table S3 for a complete list of all categorical variables and their levels. The disturbance variable included agriculture, eutrophication, hydrological disruption, logging, mining, and oil spills, or multiple of those categories. The ecosystem variable included forest, freshwater wetland, grassland, lake, mangrove, marine, river, and tidal wetland. The metric variable included abundance, animal health, carbon, community characteristic, diversity, morphology, nitrogen, nutrient, organic matter, phosphorus, soil characteristics, and water characteristics (see Table S2 for examples). The organism variable included alga/bryophyte, bacterium, bird/fish, decomposer, fungus, grass/herb, invertebrate, mammal, protozoa, subaquatic vegetation, and tree/shrub.

Time to recover and scale. Variables ranged in the time that they had to recover from less than 1 to 900 years (median = 10 years). Of the 2,820 variables for which the scale of disturbance was reported, it averaged 2,290km^2 (median 10km^2) and for the 1,257 variables for which the scale of study area was measured, it averaged 130km^2 (median 0.11km^2). While study areas were generally smaller than the scale of disturbance, they were still sizable compared to plot-scale studies and so our data represent large-scale responses to disturbance.

Choices of reference. The choice of a reference has the potential to influence answers about recovery. For example, if pre-disturbance data from times far in the past are used as restoration goals, disturbances in the intervening years including climate change may make such goals unrealistic and unattainable. In our dataset, 1092 variables compared recovery against pre-disturbance references versus 4050 variables that used a contemporaneous reference site, minimizing the impact that pre-disturbance data had on the outcomes measured here. We also found only 114 variables that used pre-disturbance controls were for disturbances that occurred prior to 1970, which we consider to be a potentially unattainable reference goal. Therefore, the vast majority of the data in this study (79%) used contemporaneous references and of those using pre-disturbance references, 978 (89%) were for contemporary disturbances. Furthermore, if the use of pre-disturbance references were to influence the results because climate change or other factors make those goals unattainable, we would expect to see variables using those references taken prior to 1970 to be further from complete recovery (i.e. have lower response ratios) and slower recovery rates. In fact, we found no difference in either recovery completeness or recovery rates for studies prior to and after 1970 ($p=0.25$, 0.34, respectively; See Electronic supplementary material, Fig. S5). Taken together, these data indicate that the choice of reference goal is not responsible for the patterns our study shows.

References:

1. Moreno-Mateos D, Power ME, Comín FA, & Yockteng R (2012) Structural and Functional Loss in Restored Wetland Ecosystems. *PLoS Biol* 10(1):e1001247.
2. Rey-Benayas JM, Newton AC, Diaz A, & Bullock JM (2009) Enhancement of biodiversity and ecosystem services by ecological restoration: A meta-analysis. *Science* 325(5944):1121-1124.
3. Meli P, Rey-Benayas JM, Balvanera P, & Ramos MM (2014) Restoration enhances wetland biodiversity and ecosystem service supply, but results are context-dependent: a meta-analysis. *PloS one* 9(4):e93507.
4. Viola DV*, et al.* (2010) Competition–defense tradeoffs and the maintenance of plant diversity. *Proceedings of the National Academy of Sciences* 107[13]:17217-17222.
5. Viechtbauer W (2010) Conducting meta-analyses in R with the metafor package. *J Stat Softw* 36[14]:1-48.
6. R Core Team (2013) A language and environment for statistical computing. R Foundation for Statistical Computing, Vienna, Austria. ISBN 3-900051-07-0, URL http://www.R-project.org/.

7. Crawley MJ (2012) *The R book* (John Wiley & Sons).

8. Lajeunesse MJ (2009) Meta‐Analysis and the Comparative Phylogenetic Method. *The American Naturalist* 174[14]:369-381.

9. Gurevitch J, Curtis PS, & Jones MH (2001) Meta-analysis in ecology. *Advances in Ecological Research* 32:199-247.

10. Wickham H (2009) *ggplot2: elegant graphics for data analysis* (Springer Science & Business Media).

11. Rosenberg MS, Hannah R R, Gurevitch J, Rothstein HR, & Jurevitch J (2013) Effect Sizes: Conventional Choices & Calculations. *Handbook of meta-analysis in ecology and evolution*, eds Koricheva J, Gurevitch J, & Mengersen K (Princeton University Press), pp 61-71.

12. Mengersen K, Schmid CH, Jennions MD, & Gurevitch J (2013) Statistical Models and Approaches to Inference. *Handbook of meta-analysis in ecology and evolution*, eds Koricheva J, Gurevitch J, & Mengersen K (Princeton University Press), pp 89-107.

13. Moher D, Liberati A, Tetzlaff J, & Altman DG (2009) Preferred reporting items for systematic reviews and meta-analyses: the PRISMA statement. *Annals of internal medicine* 151(4):264-269.

Supplementary Figures

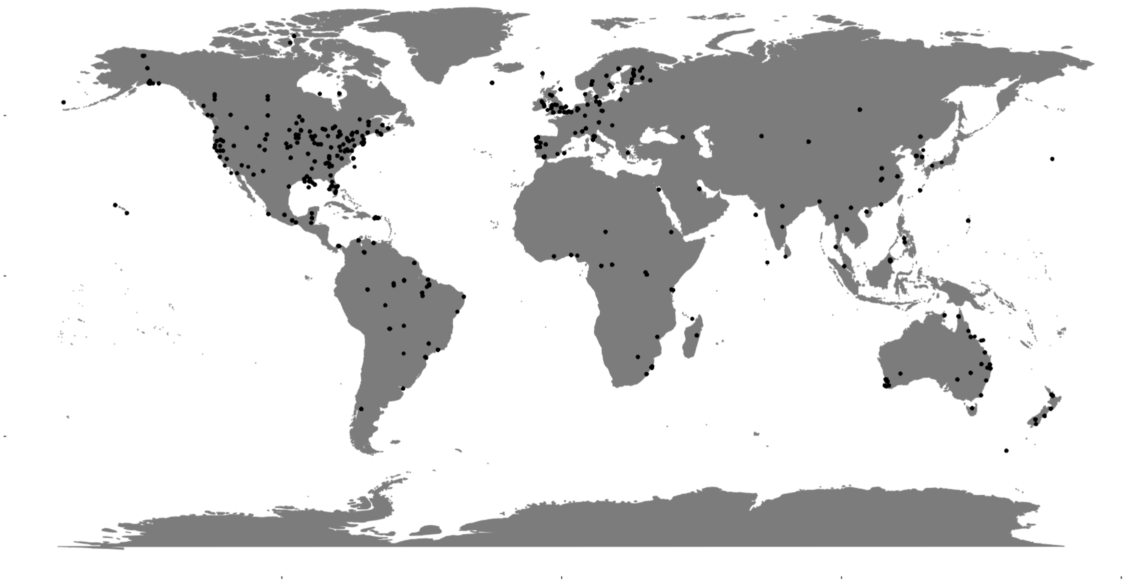

Figure S1. Geographic locations of studies included in the meta analysis.

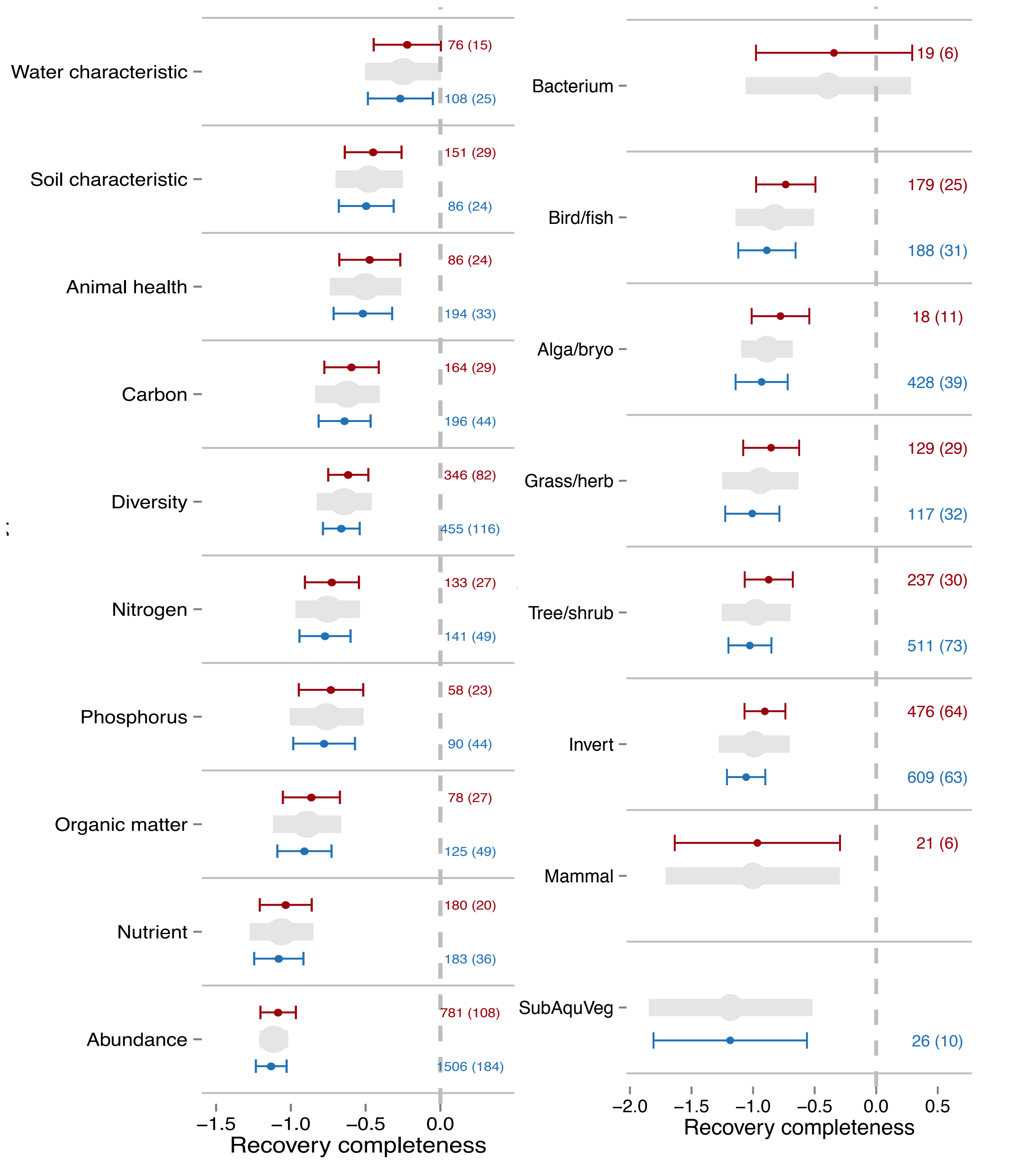

Figure S2. Recovery completeness ± 95% confidence intervals of variables categorized by metric type (left) and organism type (right) in actively restored (red) and passively recovering (blue) systems. See legend of Fig. 2 for interpretation complete recovery, difference among data points, order of data, and numbers on the sides.

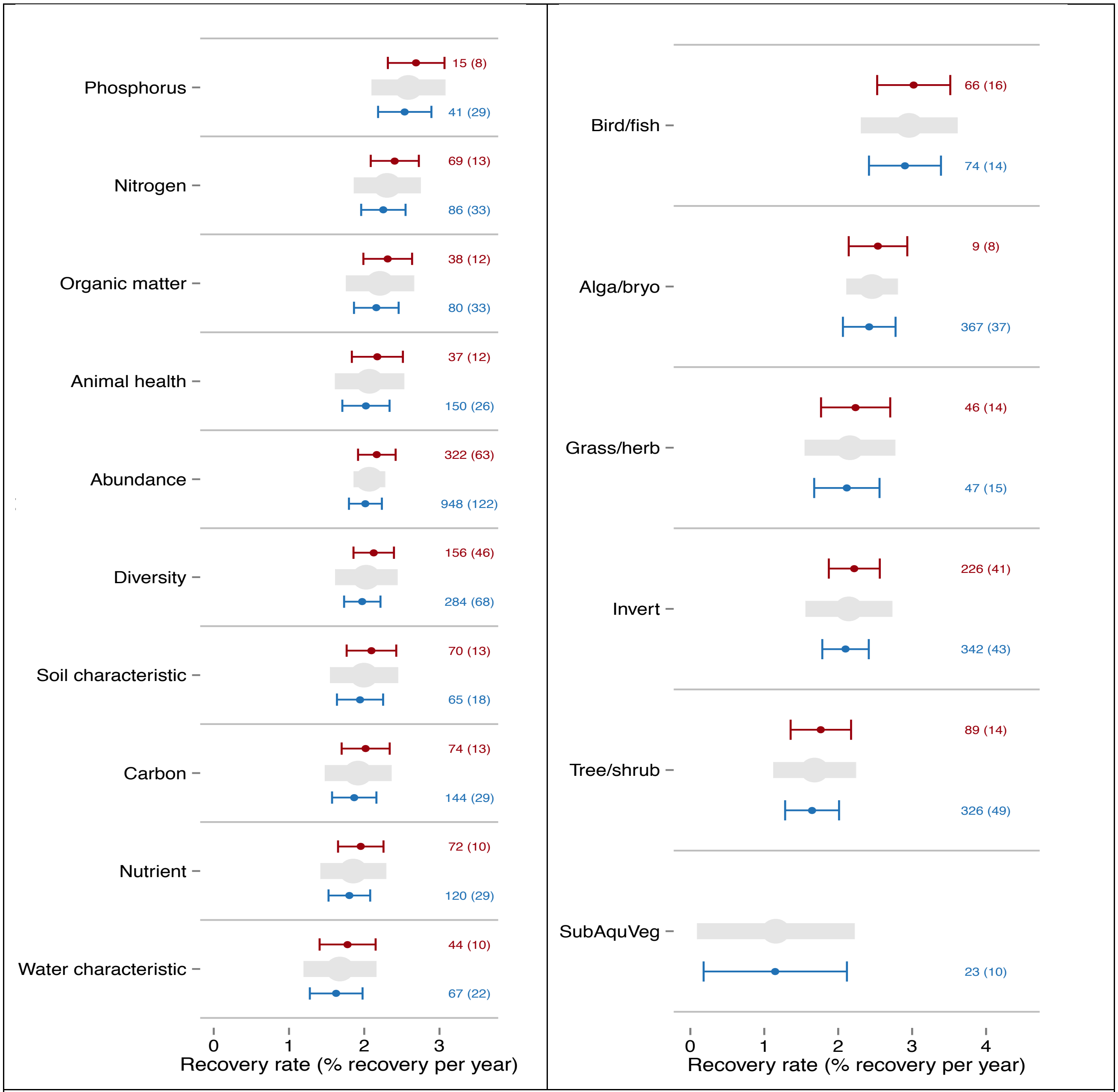

Figure S3. Recovery rate ± 95% confidence intervals of metric types (left) and organism type (right) in actively restored (red) and passively recovering (blue) systems. See legend of Fig. 2 for interpretation of difference among data points, order of data, and numbers on the sides.

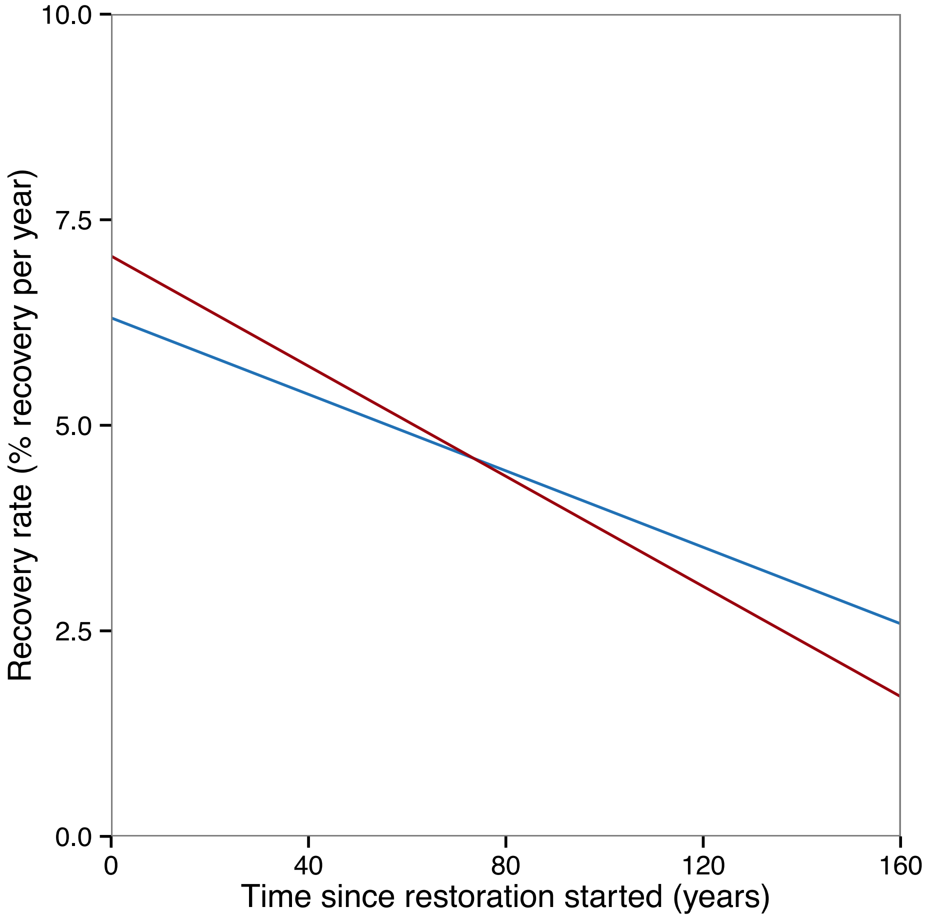

Figure S4. Modeled recovery rates decrease with time since restoration. Blue line is for passively recovering variables and red line is for actively restored variables.

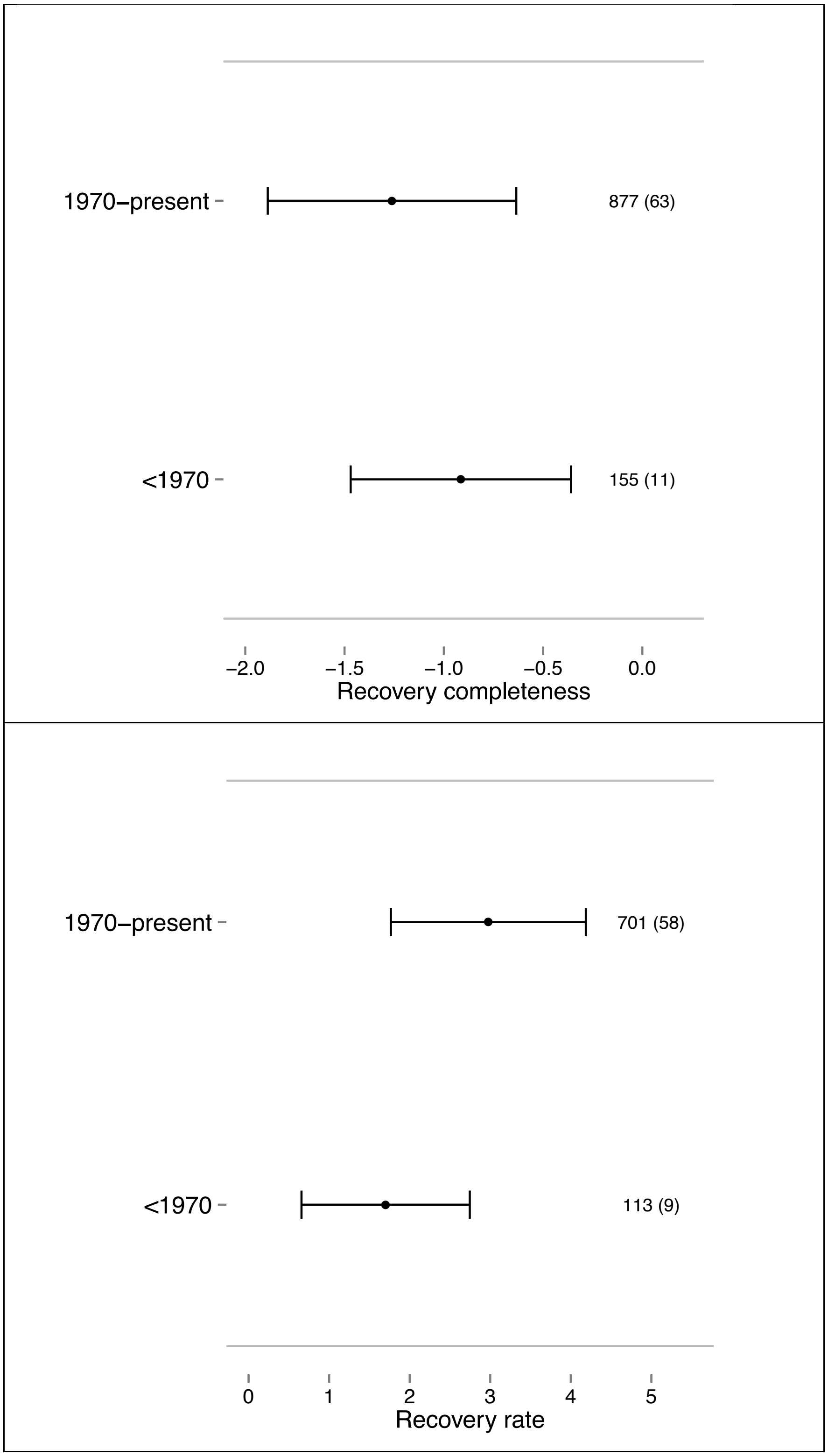

Figure S5. Boxplot of recovery completeness (top) and recovery rate (bottom) ± 95% confidence intervals for studies that used pre-disturbance controls measured before and after 1970.

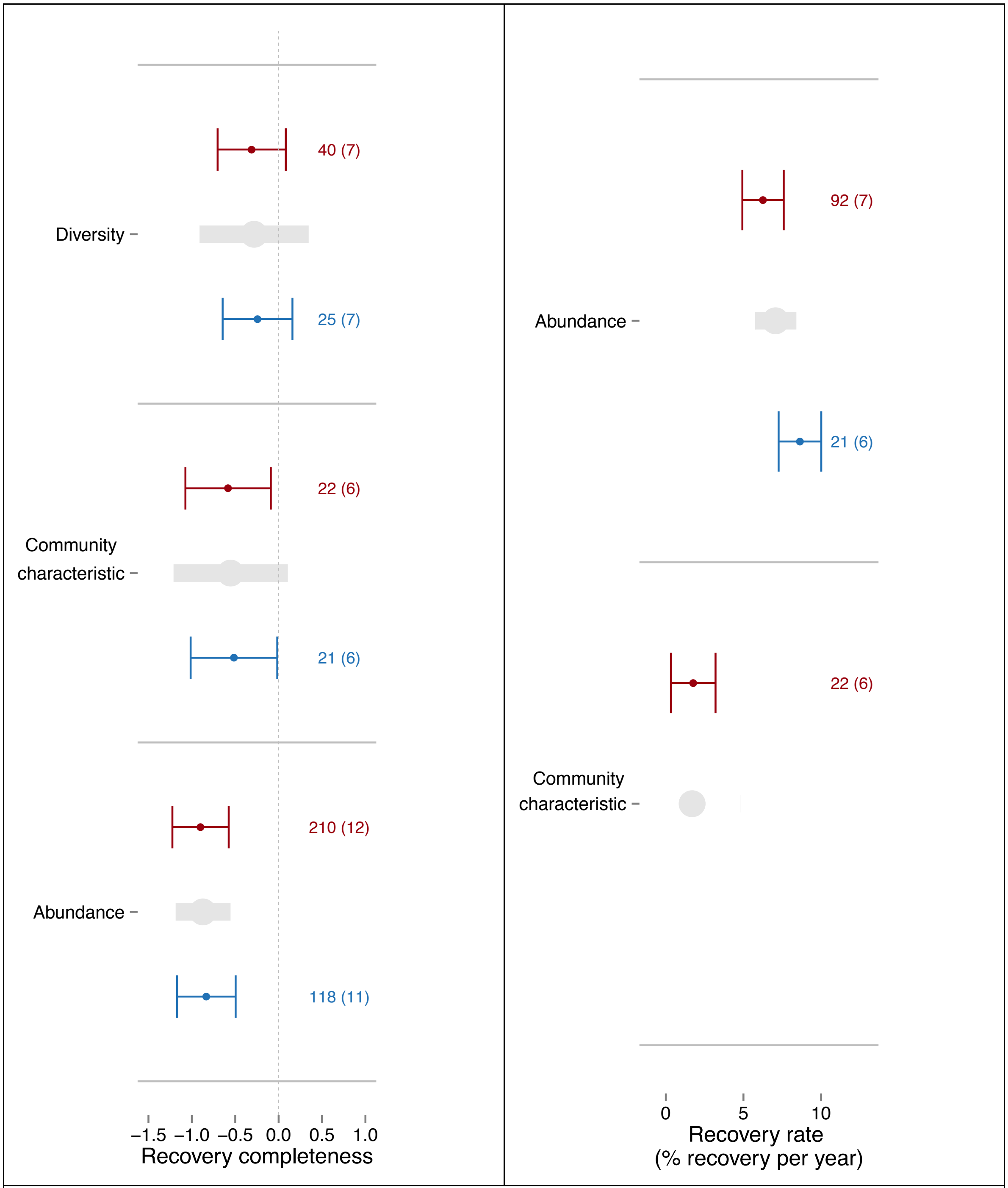

Figure S6. Recovery completeness $\pm$ 95% confidence intervals of metric types for the passive-active only dataset – those studies that directly compared active restoration with passive recovery in the same location after the same disturbance (left) and recovery rate $\pm$ 95% confidence intervals of metric types for the passive-active only dataset (right) for actively restored (red) and passively recovering (blue) systems.

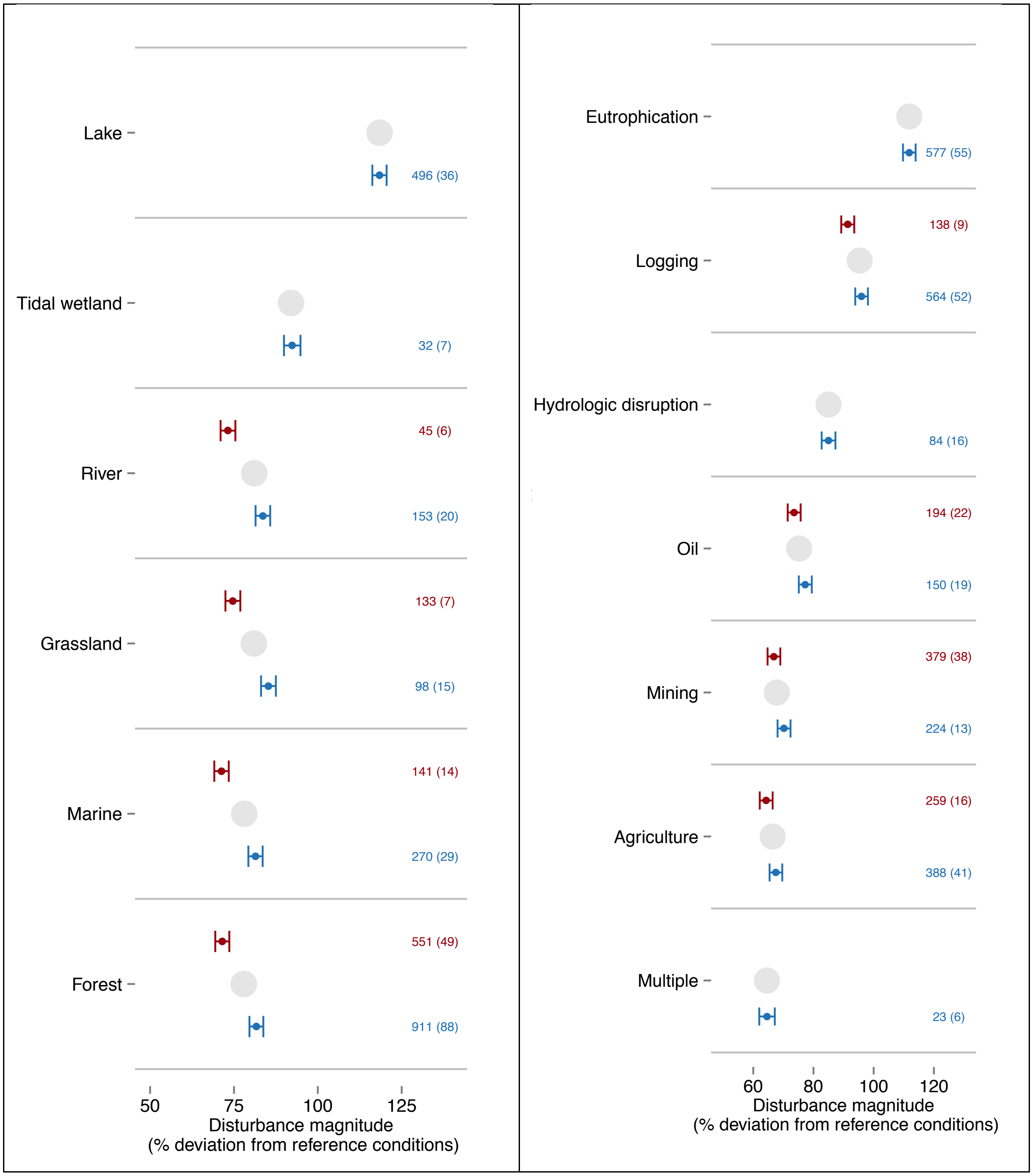

Figure S7. Disturbance magnitude ± 95% confidence intervals of variables categorized by ecosystem type (left) and disturbance type (right) in actively restored (red) and passively recovering (blue) systems. See legend of Fig. 2 for interpretation of complete recovery, difference among data points, order of data, and numbers next to lines.

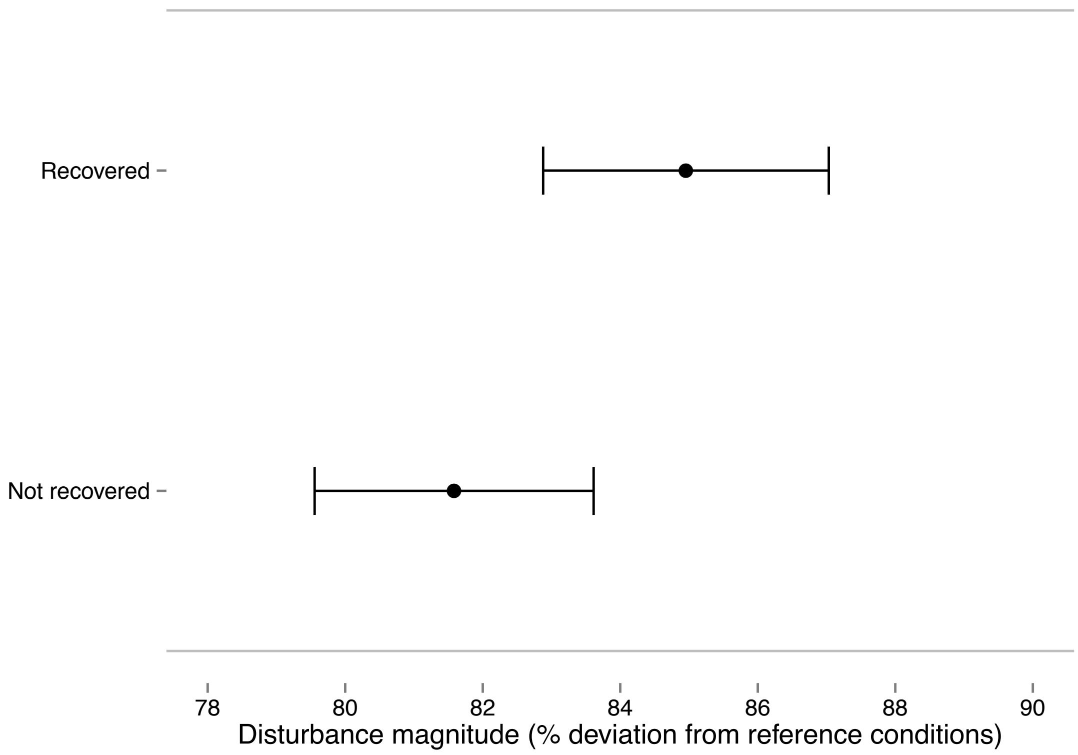

Figure S8. Mean disturbance magnitude ± 95% confidence intervals, categorized by whether variables had completely recovered (≥0.95 recovery completeness).

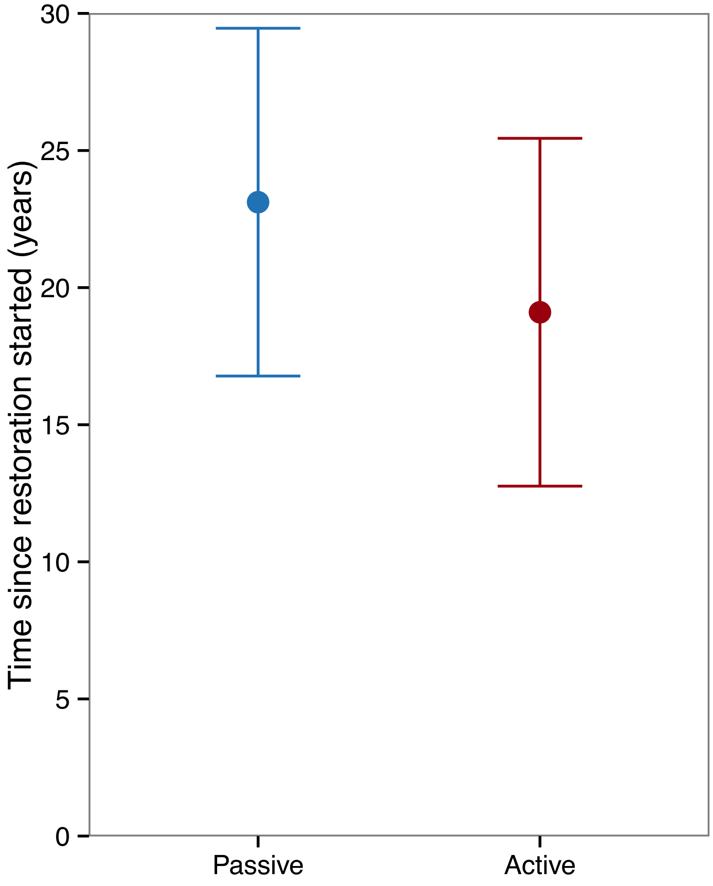

Figure S9. Mean time since restoration ± 95% confidence intervals, categorized by restoration action.

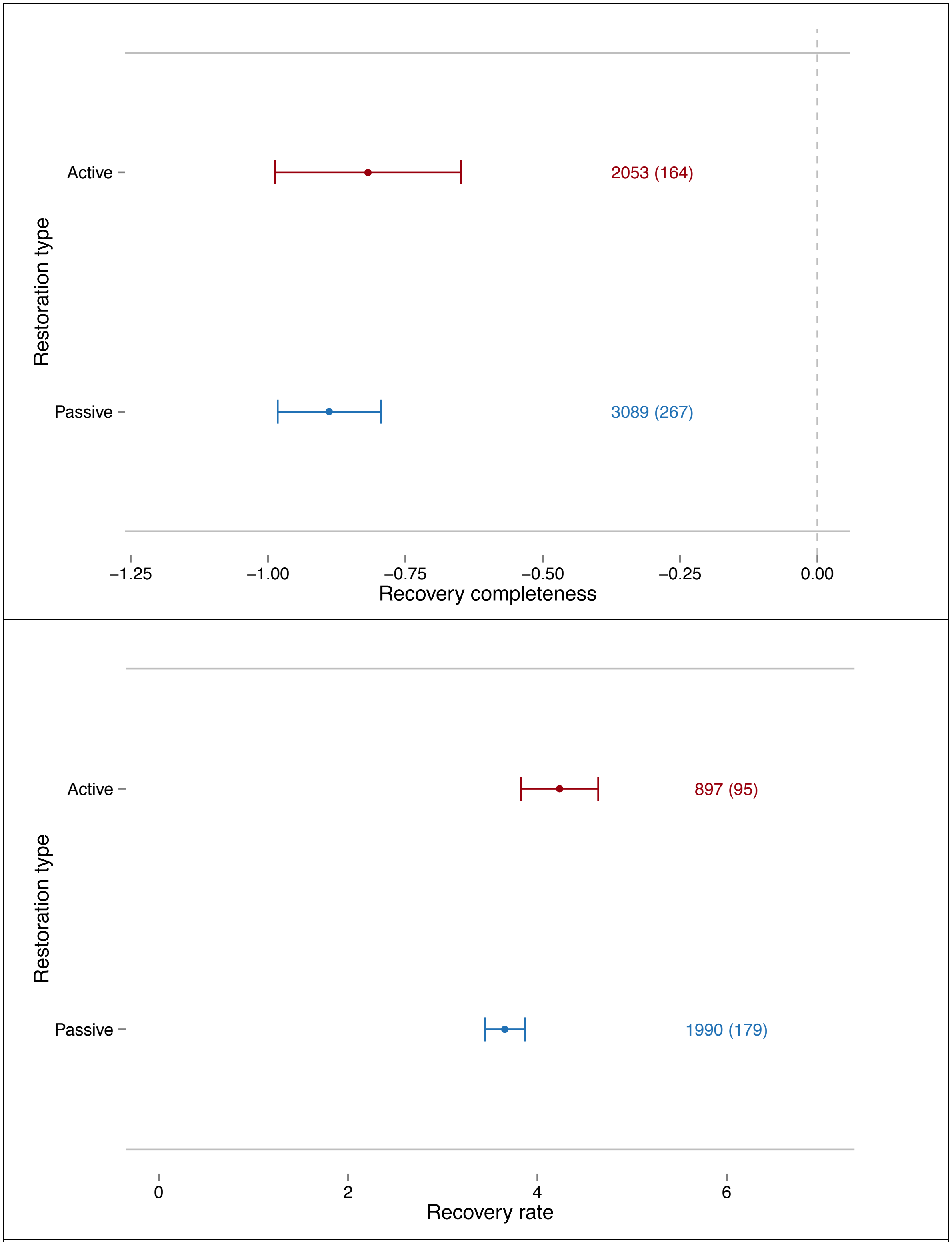

Figure S10. Recovery completeness ± 95% confidence intervals (top) and recovery rate ± 95% confidence intervals of all data categorized only by restoration action.

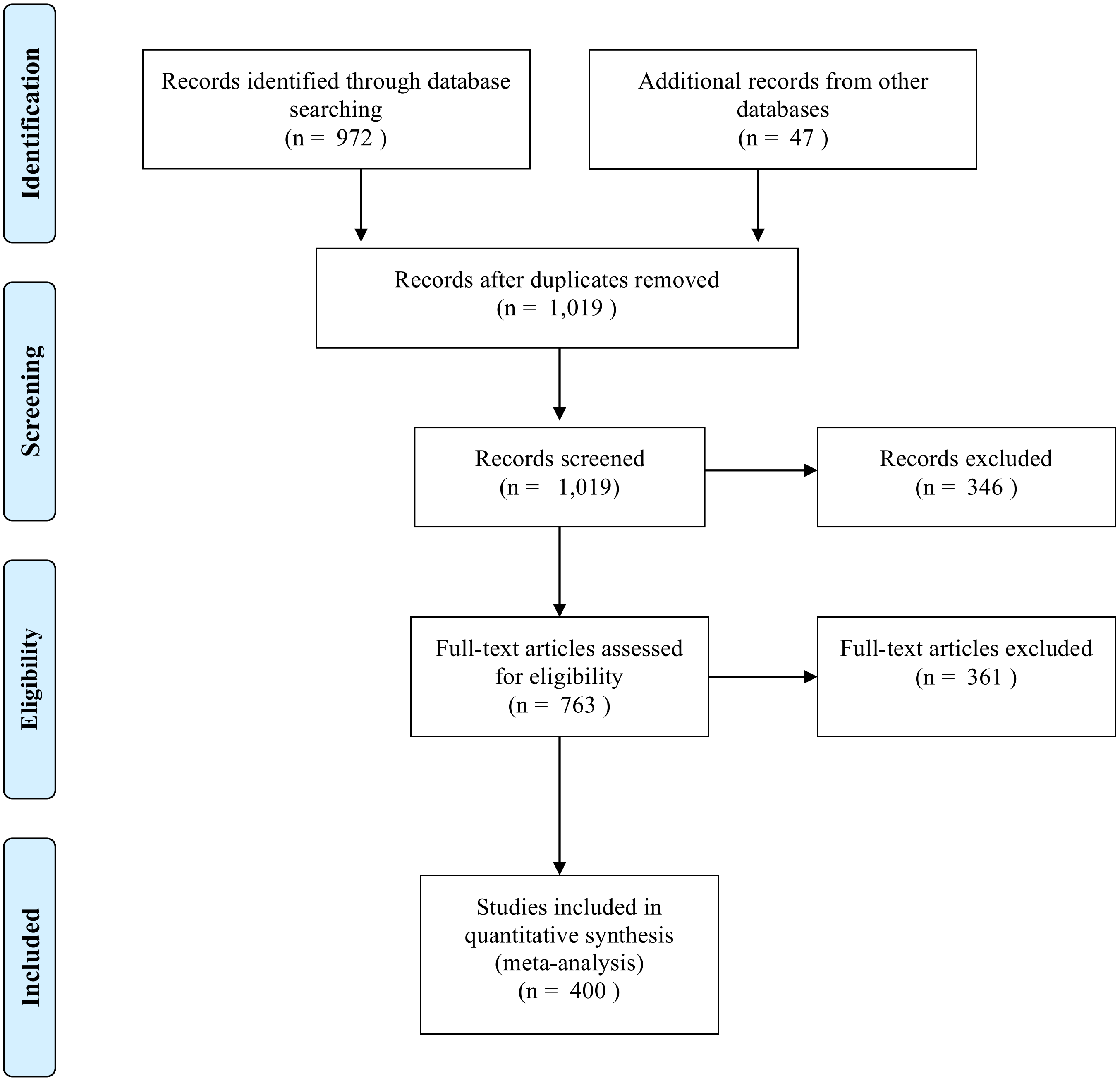

Figure S11. Prisma flow chart of studies included in the meta analysis. Structure and template for flow chart from [13].

Supplementary Tables

Table S1. Examples of active restoration and passive recovery.

	**Examples**
**Active Restoration**	Alum application
	Applying fertilizer
	Applying oil dispersants
	Artificial reef building
	Creating wetland/pond
	Flushing lakes
	Inserting woody debris
	Planting desired species mix
	Prescribed fire
	Recontouring/dredging
	Remediation of mine-polluted waters
**Passive Recovery**	Ending agricultural activities, logging, mining, oil spills, etc.
	Reconnecting waterways to reestablish wetlands
	Removing dams

Table S2. Metric types used to categorize response variables along with the most common measures of each metric type.

**Metric type**	**Most common examples**
Abundance	Biomass, density, number of individuals, percent cover, relative abundance
Animal health	Hormone levels, number injured, pollutant content in tissues
Carbon	Concentration, content, decomposition, flux, respiration
Community characteristic	Ecosystem cycling measures (NPP, NEP, GPP), importance values, relative abundance of indicator species, similarity/dissimilarity indices
Diversity	Evenness, Simpson's index, Shannon's index, species/family/genera richness
Physical structure	Area, depth, roughness, width
Nitrogen	Ammonium, inorganic, mineralization, nitrate, nitrification, organic
Nutrient	Any nutrient other than C, N, and P (e.g. Ca, Mg, Na) not in either soil or water
Nutrient ratio	C:N, Fe:P
Organic matter	Constituents of organic matter, relative abundance of constituents in organic matter, soil organic matter
Phosphorous	Available, Bray, organic, phosphate, reactive, total
Soil characteristic	Bulk density, cation exchange capacity, constituents of soil (composition), depth, moisture content, soil nutrients (other than C, N, or P), pH, temperature
Water characteristic	Clarity, depth, flow rate, pH, sedimentation rate, temperature, velocity, turbidity, water nutrients (other than C, N, or P)

Table S3. Factors used as categorical variables across all models and their levels.

**Category**	**Levels**
Disturbance type	• Agriculture<br>• Eutrophication<br>• Hydrological disruption<br>• Logging<br>• Mining<br>• Oil spill
Ecosystem type	• Forest<br>• Freshwater wetland<br>• Grassland<br>• Lake<br>• Mangrove<br>• Marine<br>• River<br>• Tidal wetland
Metric type	• Abundance<br>• Animal health<br>• Carbon<br>• Community characteristic<br>• Diversity<br>• Morphology<br>• Nitrogen<br>• Nutrient<br>• Organic matter<br>• Phosphorus<br>• Soil characteristic<br>• Water characteristic
Organism type	• Alga/bryophyte<br>• Bacterium<br>• Bird/fish<br>• Decomposer<br>• Fungus<br>• Grass/herb<br>• Invertebrate<br>• Mammal<br>• Protozoa<br>• Subaquatic vegetation<br>• Tree/shrub

Table S4. Data sets, response variables, categorical variables, sample sizes, and figure showing model results (when included) for each model run. Each row denotes a separate model.

	Data set	Dependent variable	Moderator variables	N	Figure
1	Passive-active	Recovery completeness	Metric type + recovery type	436	S6
2			Metric type	436	S6 (grey bars)
3		Recovery rate	Metric type + recovery type	135	S6
4			Metric type	135	S6 (grey bars)
5	All-studies	Recovery completeness	Disturbance type + recovery type	5,142	2A
6			Ecosystem type + recovery type	5,142	2B
7			Metric type + recovery type	5,137	S2
8			Organism type + recovery type	4,913	S2
9			Disturbance type	5,142	2A (grey bars)
10			Ecosystem type	5,142	2B (grey bars)
11			Metric type	5,137	S2 (grey bars)
12			Organism type	4,913	S2 (grey bars)
13			Recovery type	5,142	S10
14			Disturbance end	1,032	S5
15		Recovery rate	Disturbance type + recovery type	2,887	3A
16			Ecosystem type + recovery type	2,831	3B
17			Metric type + recovery type	2,882	S3
18			Organism type + recovery type	1,615	S3
19			Disturbance type	2,887	3A (grey bars)
20			Ecosystem type	2,831	3B (grey bars)
21			Metric type	2,882	S3 (grey bars)
22			Organism type	1,615	S3 (grey bars)
23			Time since restoration started + recovery type	2,853	—
24			Time since restoration started * recovery type	2,853	S4
25			Recovery type	2,887	S10
29			Disturbance end	814	S5
30		Disturbance magnitude	Disturbance type + recovery type	2,980	S7
31			Ecosystem type + recovery type	2,830	S7
32			Disturbance type	2,980	S7 (grey bars)
33			Ecosystem type	2,830	S7 (grey bars)
34			Recovery status	3,365	S8
35		Time since restoration started	Recovery type	4,104	S9
